# Identification of Novel *Oryza sativa* miRNAs in Deep Sequencing-Based Small RNA Libraries of Rice Infected with *Rice Stripe Virus*

**DOI:** 10.1371/journal.pone.0046443

**Published:** 2012-10-10

**Authors:** Weixia Guo, Gentu Wu, Fei Yan, Yuwen Lu, Hongying Zheng, Lin Lin, Hairu Chen, Jianping Chen

**Affiliations:** 1 State Key Laboratory Breeding Base for Zhejiang Sustainable Pest and Disease Control, Ministry of China Key Laboratory of Biotechnology in Plant Protection, Institute of Virology and Biotechnology, Zhejiang Academy of Agricultural Sciences, Hangzhou, China; 2 Plant Protection College, Yunnan Agricultural University, Kunming, China; 3 College of Agriculture and Biotechnology, Zhejiang University, Hangzhou, China; Ghent University, Belgium

## Abstract

MicroRNAs (miRNAs) play essential regulatory roles in the development of eukaryotes. Methods based on deep-sequencing have provided a powerful high-throughput strategy for identifying novel miRNAs and have previously been used to identify over 100 novel miRNAs from rice. Most of these reports are related to studies of rice development, tissue differentiation, or abiotic stress, but novel rice miRNAs related to viral infection have rarely been identified. In previous work, we constructed and pyrosequenced the small RNA (sRNA) libraries of rice infected with *Rice stripe virus* and described the character of the small interfering RNAs (siRNA) derived from the RSV RNA genome. We now report the identification of novel miRNAs from the abundant sRNAs (with a minimum of 100 sequencing reads) in the sRNA library of RSV-infected rice. 7 putative novel miRNAs (pn-miRNAs) whose precursor sequences have not previously been described were identified and could be detected by Northern blot or RT-PCR, and were recognized as novel miRNAs (n-miRNAs). Further analysis showed that 5 of the 7 n-miRNAs were up-expressed while the other 2 n-miRNAs were down-expressed in RSV-infected rice. In addition, 23 pn-miRNAs that were newly produced from 19 known miRNA precursors were also identified. This is first report of novel rice miRNAs produced from new precursors related to RSV infection.

## Introduction

MicroRNAs (miRNAs) are small 19–24 nt RNAs that play essential roles in eukaryotes by targeting complementary mRNAs for degradation or translational repression [1], [2]. In plants, primary miRNA (pri-miRNA) is first transcribed by polymerase II, and then processed by Dicer-like 1 (DCL1) into the precursor miRNA (pre-miRNA), normally of about 70–300 nucleotides (nt). The pre-miRNA is further processed into the mature miRNA:miRNA* duplex [3], [4], [5]. These processes occur in the nucleus. In the next stage, the duplex is transferred into the cytoplasm and unwound [3], [4]. The miRNA is then assembled into and RNA-induced silencing complex (RISC) and guides the RISC to cleave or suppress the target mRNA [3], [4], [6].

miRNAs in plants regulate leaf morphogenesis, the development of roots and flowers and other key processes, and are recognized as important regulators of plant development [7], [8], [9], [10], [11], [12]. Recent research has revealed that miRNAs also play roles in plant defense against pathogens by regulating the expression of resistance (R) genes directly or indirectly, or targeting the viral genome to impair viral replication [13], [14], [15], [16], [17]. Hence, the miRNA pathway also plays a key role during pathogen-plant interactions.

In plants, over 4600 miRNAs have been identified from over 50 species (miRBase version 18.0, http://www.mirbase.org/cgi-bin/browse.pl) [18]. *Medicago truncatula*, *Oryza sativa* and *Glycine max* are the three plants that have the most identified miRNAs (respectively 674, 661 and 395 miRNAs) [18]. Some miRNA families have functions that are conserved across the plant kingdom and thus their sequences are similarly conserved (e.g. miR156, miR159, miR160 and miR165). Other miRNA families are specific to particular plants, and are not found elsewhere, indicating that they have novel and specific functions [19], [20].

With the development of next generation sequencing technologies, deep-sequencing has provided a powerful high-throughput strategy for identifying novel miRNAs. In this way, hundreds of miRNAs have been identified from *Arabidopsis*, *Brassica rapa*, rice, wheat, barley, peanuts, grapevine and other plants [21], [22], [23], [24], [25], [26], [27], [28], [29], [30], [31], [32]. In rice, Sunkar et al identified 23 new miRNAs from three small RNA (sRNA) libraries of control rice seedlings and seedlings exposed to drought or salt stress; six of the new miRNAs are conserved in monocots [27]. Chen et al identified 24 novel microRNA families from rice embryogenic callus, some of which were suggested to function in meristem development [33]. Li et al investigated the H_2_O_2_-regulated miRNAs in rice seedlings and discovered 32 new miRNAs [34]. Peng et al identified 43 novel miRNAs from the sRNA libraries of rice spikelets [35], while Wang et al identified 75 novel miRNAs from the developing pollen of rice [36].

Until now, most reports of novel rice miRNAs have been related to studies of rice development, tissue differentiation, or abiotic stress, while the novel rice miRNAs related to viral infection have rarely been identified. Identifying the novel miRNAs related to viral infection in rice would be helpful to broaden our understanding of the miRNA response of rice to virus infection in general and is likely to provide a model for other cereal plants in particular. *Rice stripe virus* (RSV) is the type member of the genus *Tenuivirus*. It is transmitted by the brown planthopper *Laodelphax striatellus* in a persistent manner and causes rice stripe disease, which is severe in rice fields in East Asia [37]. In a recent study, Du et al reported the changed expression of the known miRNAs during the RSV infection, and found that the known osa-miR168, 156, 396, 159, 535, 166, 172, 167, 528 and 444 were the ten most abundant miRNA families in RSV-infected rice, and that RSV infection induced the expression of novel miRNAs in a phased pattern from several conserved miRNA precursors, and enhanced the accumulation of some rice miRNA*s, but not their corresponding miRNAs [38].

In previous work, we constructed and pyrosequenced sRNA libraries of rice inoculated with RSV and mock-inoculated controls, and described the character of the small interfering RNAs (siRNA) derived from the RSV RNA genomes [39]. Here, we report the identification of novel miRNAs from the abundant sRNAs (with a minimum of 100 sequencing reads) in the reported sRNA library of RSV-infected rice. 7 putative novel miRNAs (pn-miRNAs) whose precursor sequences have not been described before were identified. Transcription analysis revealed that 7 pn-miRNAs were produced in rice, and that their expression levels changed in RSV-infected rice. This is first report on novel rice miRNAs produced from new precursors related to RSV infection, and will deepen our understanding of miRNA functions during RSV infection.

## Materials and Methods

### sRNA libraries used here

In our previous work, we constructed sRNA libraries of RSV-infected and non-infected rice by the Illumina Solexa sequencing system [39]. A total of 917,776 and 1,009,009 unique sequences of 18–30 nt long small RNAs were contained in the respective sRNA libraries of RSV-infected and non-infected rice. About 23% of within these two sets was similar. Almost 40% of sequences were 24 nt long, 23% of sequences were 21 nt and 15% were 22 nt [39]. Here, we attempted to identify novel miRNAs in the RSV-infected sRNA library. For optimum identification and detectability by gel blot in the following experimental confirmation, we only chose to analyze the highly abundant sRNAs (total 239 sequences) that had a minimum of 100 sequencing reads (Table S1). Three sRNA libraries of RSV-infected rice reported by Du et al were also used to search for the putative miRNAs identified here [38].

### Identification of putative novel miRNAs

The analysis was done in three steps. In the first step, we aligned the 239 chosen sRNAs with all the mature miRNAs in miRBase to identify the known miRNAs. The recognized miRNA sequences in sRNAs were excluded from the next step. In the second step, the remaining sRNAs were aligned with all the reported miRNA precursor sequences to identify putative miRNAs newly produced from the known precursors. These sRNAs were excluded from the following step. In the third step, the precursor sequences of all remaining sRNAs were predicted using a modification of the reported method [27]. Briefly, searches were made for the sRNAs in the rice genomic sequences (http://www.ncbi.nlm.nih.gov/blast/Blast.cgi) and their loci in the genome were recorded. The sRNAs that had more than 26 loci in the rice genome were not considered in the following analysis for two reasons: 1) the highest number of loci of known miRNAs in the rice genome is 26 (osa-miR395); and 2) the higher number of loci more often occurs when the loci are located in the repeat-rich regions of the rice genome; siRNAs but not miRNAs can be produced from the repeat-rich regions that have predicted fold-back structures (miRBase). This largely excludes the contaminative effect of siRNA on miRNA identification.

For each locus, two sequences respectively extending 200 nt upstream and 20 nt downstream, or 20 nt upstream and 200 nt downstream of the sRNA were extracted for secondary structure prediction. The secondary structures were predicted by the Mfold RNA folding platform (http://mfold.rna.albany.edu/?q=mfold/RNA-Folding-Form). A sequence that has a secondary structure with at least 16 paired nucleotides in its stem region and has free energy less than or equal to −20 kCal/mol was considered to be a putative precursor sequence.

### Plant materials

RSV-infected rice were prepared as described [39]. Briefly, viruliferous adult brown planthoppers (*Laodelphax striatellus* Fallen) (carrying the RSV-Zhejiang isolate) were transferred onto healthy rice seedlings (*Oryza sativa* L. japonica. cv. Nipponbare) at the three-leaf stage for virus inoculation. Control seedlings were inoculated with non-viruliferous planthoppers. After 72 h, the planthoppers were removed. Systemic infections were confirmed by RT-PCR specific for RSV Zhejiang isolate [40]. One week after inoculation, leaves were collected from the infected and control (Mock) plants, frozen and stored at −80°C until used. All rice plants were grown in a glasshouse at 28–30°C day/25°C night, with a 12 h day/night light cycle under well-watered conditions.

### sRNAs gel blot analysis

Total RNA was isolated from the frozen plant materials with Trizol (Invitrogen, USA) according to the manufacturer's instructions. 50 µg of DNase-treated total RNA was separated on a 15% polyacrylamide gel, and transferred electrophoretically to Hybond-N+ membranes (Amersham Bioscience) using 20×SSC. Membranes were baked at 80°C for 2 hours. DNA oligonucleotides complementary to the putative miRNA sequences were end-labeled with DIG using the DIG Oligonucleotide 3′-end labeling Kit (Roche). Membranes were pre-hybridized for at least 1 h and hybridized overnight at 42°C using DIG High Prime Labeling and Detection Starter Kit II (Roche). The hybridization signals were visualized by exposure to X-ray film (Kodak).

### Reverse transcription (RT)-PCR and real-time PCR for sRNAs

We used the published method for RT-PCR of sRNAs with modifications [41]. Briefly, for each sRNA, the specific stem-loop RT primer was used for reverse transcribing from purified total RNA. Then the RT product was used for PCR with the specific forward primer and the universal reverse primer. For SYBR green-based real-time PCR analysis, the reactions were incubated in a 384-well plate at 95°C for 10 min, followed by 40 cycles of 95°C for 15 s and 60°C for 1min. U6 was used as the internal control. All reactions were run in triplicate, and the results were analyzed by the ΔΔC_T_ method. The primers used are listed in Table S2.

### Target prediction

Putative targets for the novel miRNAs were predicted by psRNATarget (http://plantgrn.noble.org/psRNATarget/) [42], using the DFCI Oryza sativa gene index release 18.0 as a reference set. The parameters for prediction were default, namely, maximum expectation was set at 3.0; length for complementarity scoring was set at 20 bp; target accessibility (range 0–100, less is better) was set at 25.0; Flanking length around target site for target accessibility analysis was set at 17 bp upstream and 13 bp downstream.

### Statistical analysis

The number of reads of each sRNA from RSV-infected rice was divided by the number of reads in the non-infected controls of the same experiment. A two-tailed *t*-test was used to evaluate whether the means of these ratios from several experiments differed significantly from 1 (equal numbers of reads).

## Results

### Identification of known miRNAs

**Figure 1:**
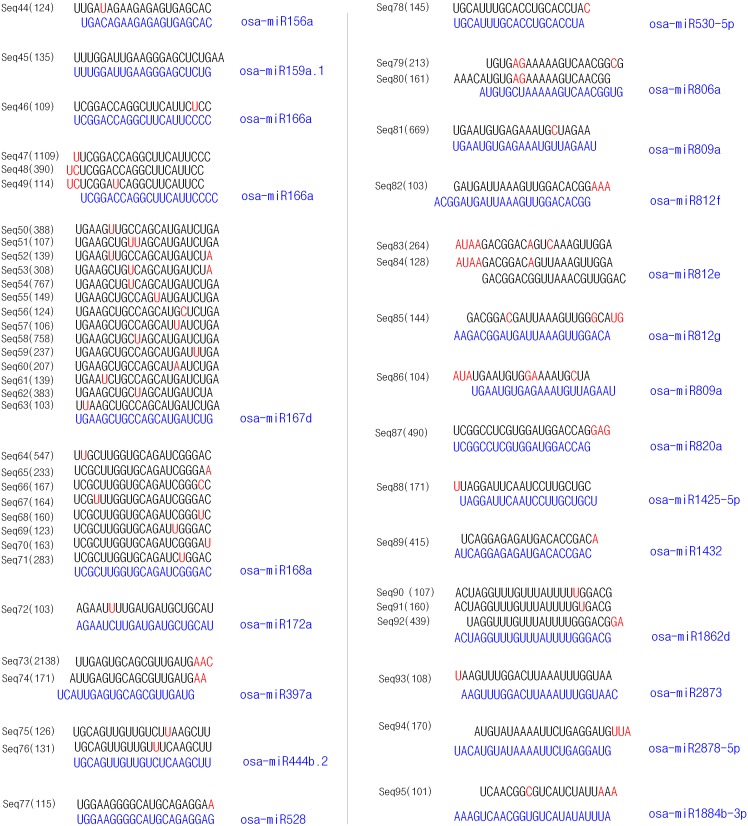
Alignment between sRNAs (names and sequences in black) that did not completely match known rice miRNAs with their closest miRNAs (names and sequences in blue). Mismatched nucleotides are shown in red.

To identify the novel miRNAs from the library, we chose 239 sRNAs that had a minimum of 100 sequencing reads for analysis according to the method reported by Sunkar et al [27]. By searching in the miRBase database (Version 18.0, http://www.mirbase.org/index.shtml), 43 sRNAs that completely matched with the deposited rice miRNAs were identified (Table 1). Meanwhile, 52 sRNA that did not completely match with the deposited rice miRNAs but contained identical or similar (>85% identity) seed sequences to the deposited rice miRNAs were also found (Figure 1). Considering that the isoforms of miRNAs exist more often in pyrosequenced sRNAs 43,44,45, we here recognized these 52 sRNAs as known rice miRNAs. Thus, a total of 95 known miRNAs were identified. These miRNAs belong to 46 miRNA families, of which the five with the highest frequency in the library were osa-miR167, 168, 528, 172 and 444. Other well-known miRNA families, osa-miR 156, 169, 170 and 397 also existed in the library (Table 1 and Figure 1).

**Table 1:** Known miRNAs identified from the sRNA library of RSV-infected rice.

sRNA No.	sRNA sequence	length	Sequencing reads	The corresponding known miRNA of sRNA
Seq1	UUUGGAUUGAAGGGAGCUCUG	21	140	osa-miR159a.1
Seq2	UGCCUGGCUCCCUGUAUGCCA	21	121	osa- miR160a
Seq3	UGCCUGGCUCCCUGAAUGCCA	21	118	osa- miR160f
Seq4	UCGAUAAACCUCUGCAUCCAG	21	572	osa-miR162a
Seq5	UGGAGAAGCAGGGCACGUGCA	21	1851	osa-miR164a
Seq6	UCGGACCAGGCUUCAUUCCCC	21	3647	osa-miR166a
Seq7	UCGGACCAGGCUUCAUUCCUC	21	601	osa-miR166g
Seq8	UCGGACCAGGCUUCAAUCCCU	21	318	osa-miR166k
Seq9	UGAAGCUGCCAGCAUGAUCUGA	22	118012	osa-miR167d
Seq10	UGAAGCUGCCAGCAUGAUCUA	21	24678	osa-miR167a
Seq11	UCGCUUGGUGCAGAUCGGGAC	21	83988	osa-miR168a
Seq12	CAGCCAAGGAUGACUUGCCGG	21	738	osa-miR169b
Seq13	UAGCCAAGGAUGACUUGCCUG	21	342	osa-miR169i.1
Seq14	CAGCCAAGGAUGACUUGCCGA	21	269	osa-miR169a
Seq15	UGAUUGAGCCGUGCCAAUAUC	21	350	osa-miR171b
Seq16	AGAAUCUUGAUGAUGCUGCAU	21	9444	osa-miR172a
Seq17	UUCCACAGCUUUCUUGAACUU	21	127	osa-miR396c
Seq18	UCCACAGGCUUUCUUGAACUG	21	1662	osa-miR396d
Seq19	UGCCAAAGGAGAGUUGCCCUG	21	286	osa-miR399d
Seq20	CUGCACUGCCUCUUCCCUGGC	21	157	osa-miR408
Seq21	UGCAGUUGUUGUCUCAAGCUU	21	6725	osa-miR444b
Seq22	UGCAGUUGCUGCCUCAAGCUU	21	333	osa-miR444a
Seq23	UGUUGUCUCAAGCUUGCUGCC	21	439	osa-miR444b.1
Seq24	UGGAAGGGGCAUGCAGAGGAG	21	19789	osa-miR528
Seq25	UGACAACGAGAGAGAGCACGC	21	2553	osa-miR535
Seq26	AAGACGGAUGAUUAAAGUUGGACA	24	387	osa-miR812g
Seq27	UAUGAAUGUGGGCAAUGCUAGAAA	24	167	osa-miR819a
Seq28	UUAGAUGACCAUCAGCAAACA	21	950	osa-miR827a
Seq29	UGUAAAAUUCAUUCGUUCCAA	21	328	osa-miR1320-3p
Seq30	UAGGAUUCAAUCCUUGCUGCU	21	722	osa-miR1425-5p
Seq31	CAGCAAGAACUGGAUCUUAAU	21	414	osa-miR1425-3p
Seq32	UAAGAUAAUGCCAUGAAUUUG	21	140	osa-miR1428e-3p
Seq33	GUUGCACGGGUUUGUAUGUUGCAG	24	520	osa-miR1429-3p
Seq34	AUCAGGAGAGAUGACACCGAC	21	528	osa-miR1432
Seq35	UGGAAAGUUGGGAGAUUGGGG	21	131	osa-miR1850.1
Seq36	CUAGAUUUGUUUAUUUUGGGACGG	24	763	osa-miR1862e
Seq37	AGCUCUGAUACCAUGUUAGAUUAG	24	253	osa-miR1863
Seq38	UGCUGAAUUAGACCUAGUGGGCAU	24	114	osa-miR1870
Seq39	AAAGUCAACGGUGUCAUAUAUUUA	24	103	osa-miR1884b-3p
Seq40	UAUUUUAGUUUCUAUGGUCAC	21	194	osa-miR2871-3p
Seq41	UUCUUGUGCUGCUGAAGAGAC	21	299	osa-miR5144-5p
Seq42	AGCUUCUGACAGCUGCAGUUUCUC	24	199	osa-miR5150-5p
Seq43	UUUGAGAAGGUAUCAUGAGAU	21	163	osa-miR5542

### Identification of miRNAs newly produced from known miRNA precursors

**Figure 2:**
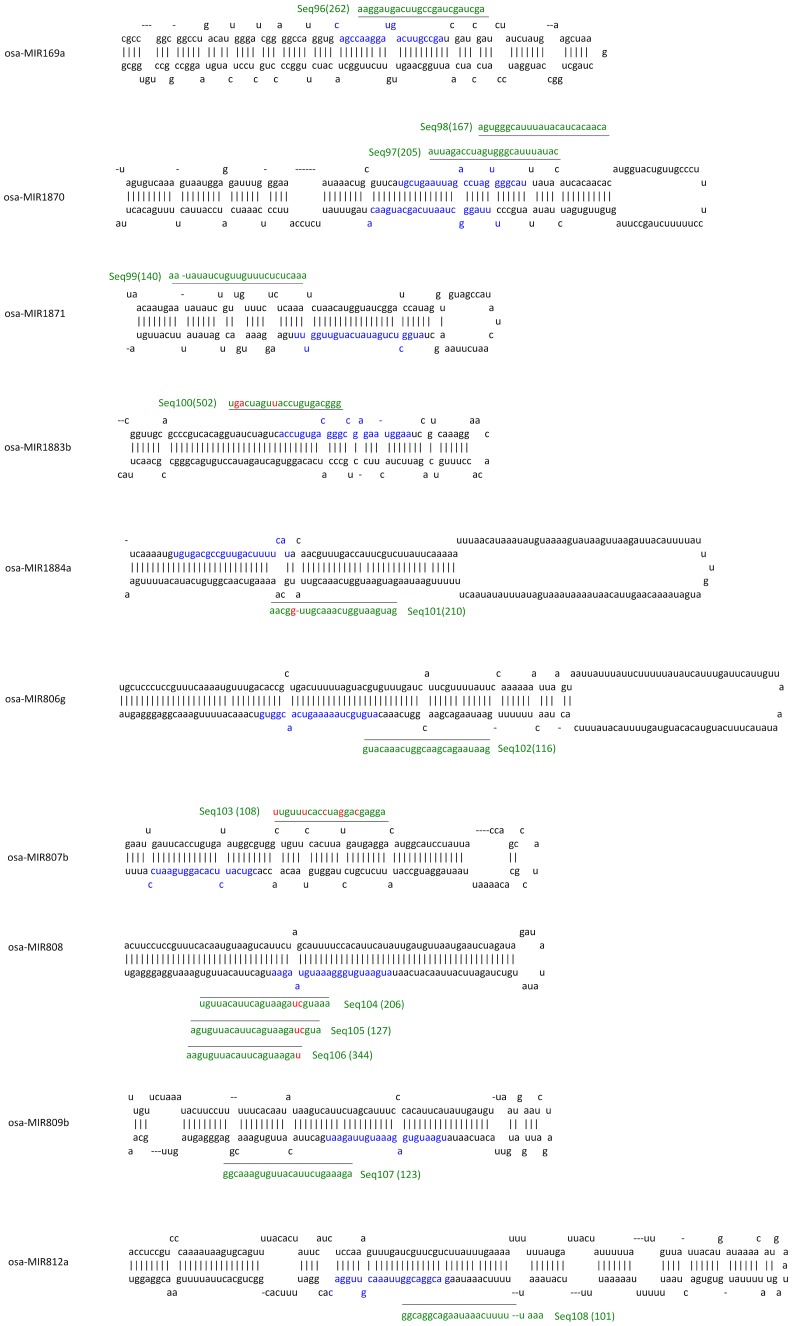
sRNAs that were recognized as newly produced miRNAs from known precursors and their location in the secondary structures of their precursors. The sequences of the known mature miRNAs in precursors are colored with blue. Names, sequences and sequencing reads (bracketed) of sRNAs identified here are colored with green. The location of sRNA is shown by a black line. Mismatched nucleotides are shown in red.

**Figure 3:**
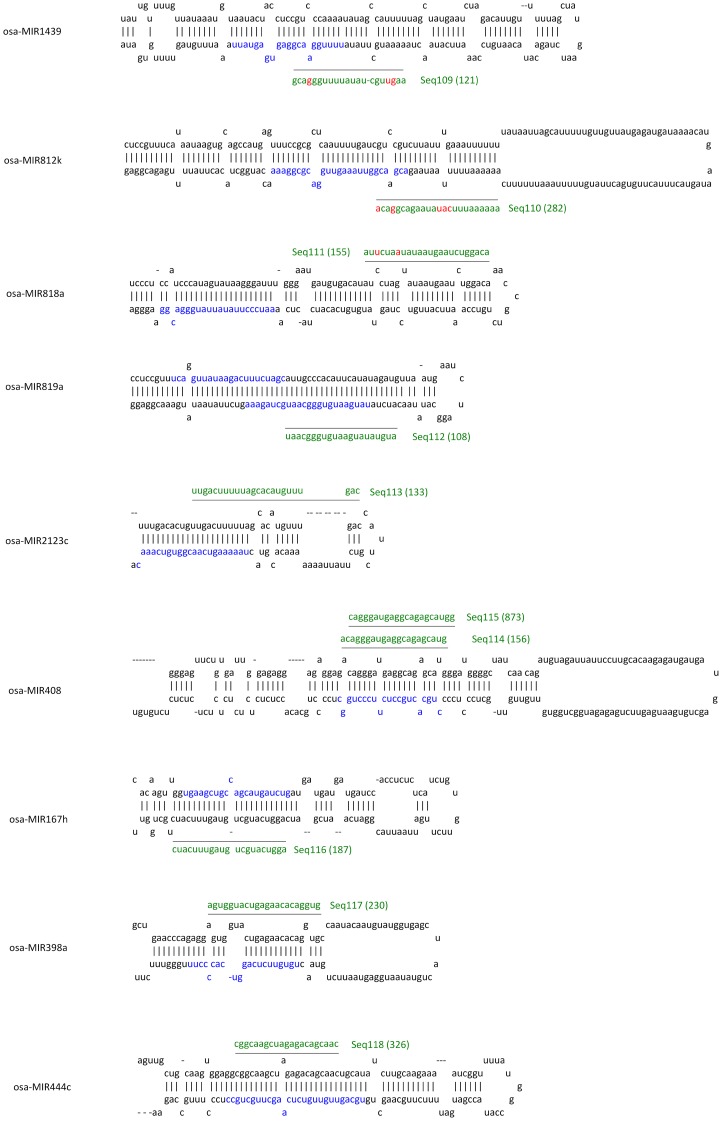
sRNAs that were recognized as newly produced miRNAs from known precursors and their location in the secondary structures of their precursors. The sequences of the known mature miRNAs in precursors are colored with blue. Names, sequences and sequencing reads (bracketed) of sRNAs identified here are colored with green. The location of sRNA is shown by a black line. Mismatched nucleotides are shown in red.

One miRNA precursor can produce several mature miRNAs with different sequences. Du et al (2011) recently reported that RSV infection induced different miRNAs to be produced from a single precursor in a phased pattern [38]. Hence, it was possible that the remaining 144 sRNAs contained some miRNAs that had been produced from a known conserved precursor but did not match known mature miRNAs. To identify these miRNAs, all 144 sRNAs were aligned with the known rice miRNA precursor stem-loop sequences. 23 of the sRNA sequences were identified among 19 known miRNA precursors, and were therefore considered to be novel miRNAs newly produced from known miRNA precursors. Their locations on the precursors are shown in Figure 2 and Figure 3.

Among these 23 sRNAs, 12 were produced from the 5′ arm and 11 were produced from the 3′ arm of their respective precursor. Seq114 and 115 were located at the miRNA* region of osa-miR408; Seq116 was located at the miRNA* region of osa-miR167 h; Seq117 was located at the miRNA* region of osa-miR398a; Seq118 was located at the miRNA* region of osa-miR444c. These sRNAs may be the miRNA* or miRNA* isoforms of the corresponding miRNAs.

We also searched for these 23 sRNA in the control library and other reported RSV and non-infected rice sRNA libraries, and found that they existed in the libraries with different sequencing reads (Table 2). Statistical analysis showed that Seq99, Seq102 and Seq107 were significantly down-expressed in RSV-infected rice, indicating that they may have a function in response to RSV infection (Table 2).

**Table 2:** The putative novel miRNAs newly produced from known miRNA precursors.

sRNA No.	sRNA sequence	Length	SR^a^ in Exp1^b^	SR in Exp2^c^	SR in Exp3^c^	SR in Exp4^c^	Mean (i/n)^d^	SD	t (3DF)	Significance (alpha)
Seq96	AAGGATGACTTGCCGATCGATCGA	24	285(231)	39(81)	0(0)	2(11)	0.63	0.542	−1.17	0.325
Seq97	ATTAGACCTAGTGGGCATTTATAC	24	223(214)	42(69)	19(128)	27(38)	0.63	0.369	−2.02	0.137
Seq98	AGTGGGCATTTATACATCACAACA	24	182(224)	36(82)	7(44)	8(6)	0.69	0.508	−1.24	0.304
Seq99	AATATATCTGTTGTTTCTCTCAAA	24	153(195)	5(14)	5(26)	9(51)	0.38	0.283	−4.39	0.022*
Seq100	TGACTAGTTACCTGTGACGGG	21	547(472)	3(18)	0(0)	0(12)	0.44	0.627	−1.54	0.221
Seq101	GATGAATGGTCAAACGTTGGACAA	24	229(207)	59(281)	17(118)	15(158)	0.39	0.481	−2.54	0.084
Seq102	GAATAAGACGAACGGTCAAACATG	24	126(153)	32(67)	20(29)	14(74)	0.54	0.277	−3.29	0.046*
Seq103	TTGTTTCACCTAGGACGAGGA	21	118(67)	14(26)	1(8)	1(16)	0.62	0.788	−0.96	0.408
Seq104	AAATGCTAGAATGACTTACATTGT	24	224(262)	32(35)	41(68)	26(335)	0.61	0.381	−2.03	0.135
Seq105	ATGCTAGAATGACTTACATTGTGA	24	138(164)	27(34)	15(38)	17(73)	0.57	0.299	−2.90	0.062
Seq106	TAGAATGACTTACATTGTGAA	21	375(350)	8(17)	6(60)	8(70)	0.44	0.455	−2.47	0.090
Seq107	AGAAAGTCTTACATTGTGAAACGG	24	134(155)	51(142)	24(75)	21(93)	0.44	0.287	−3.89	0.030*
Seq108	AAATTTTTCAAATAAGACGGACGG	24	110(81)	56(75)	2(9)	1(11)	0.60	0.577	−1.37	0.264
Seq109	AAGTTGCTATATTTTGGGACG	21	132(131)	3(7)	5(15)	4(16)	0.50	0.343	−2.89	0.063
Seq110	AAAAAATTTCATATAAGACGGACA	24	307(278)	73(64)	5(19)	4(28)	0.66	0.533	−1.26	0.295
Seq111	ATTCTAATATAATGAATCTGGACA	24	169(112)	17 (18)	0(0)	0(2)	0.82	0.762	−0.41	0.707
Seq112	ATCTATATGAATGTGGGCAAT	21	118(94)	24(20)	2(0)	7(9)	1.08	0.261	0.59	0.594
Seq113	TTGACTTTTTAGCACATGTTTGAC	24	145(133)	23(8)	1(7)	6(10)	1.18	1.196	0.30	0.787
Seq114	ACAGGGATGAGGCAGAGCATG	21	170(83)	24(30)	9(7)	11(1)	3.78	4.838	1.15	0.333
Seq115	CAGGGATGAGGCAGAGCATGG	21	951(382)	126(133)	82(42)	106(53)	1.85	0.647	2.62	0.079
Seq116	AGGTCATGCTGTAGTTTCATC	21	204(5)	37(2)	340(22)	364(63)	20.13	14.807	2.58	0.081
Seq117	AGTGGTACTGAGAACACAGGTG	22	251(101)	0(0)	2(4)	2(6)	1.11	1.197	0.15	0.888
Seq118	CGGCAAGCTAGAGACAGCAAC	21	355(355)	1800(222)	252(146)	357(121)	3.45	3.211	1.52	0.225

a: Sequencing reads (SR) were normalized to one million with the unique sequence reads of each library.

b: exp1 is the experiment reported by us. Sequencing reads in the non-infected sRNA library are bracketed, while those in the RSV-infected sRNA library are not.

c: exp2–4 are the three repeats of Du et al (2011). Sequencing reads in non-infected sRNA libraries are bracketed, while those in RSV-infected sRNA libraries are not.

d: mean of the number of infected divided by number of non-infected reads; the following columns show the standard deviation and the ***t-***test value to test whether the mean differs significantly from 1 (equal numbers of reads).

* : indicating the sRNAs that have the significant changes in RSV-infected rice (*p* value<0.05).

### Identification of putative novel miRNAs (pn-miRNAs) produced from potential new precursor sequences not previously described

**Figure 4:**
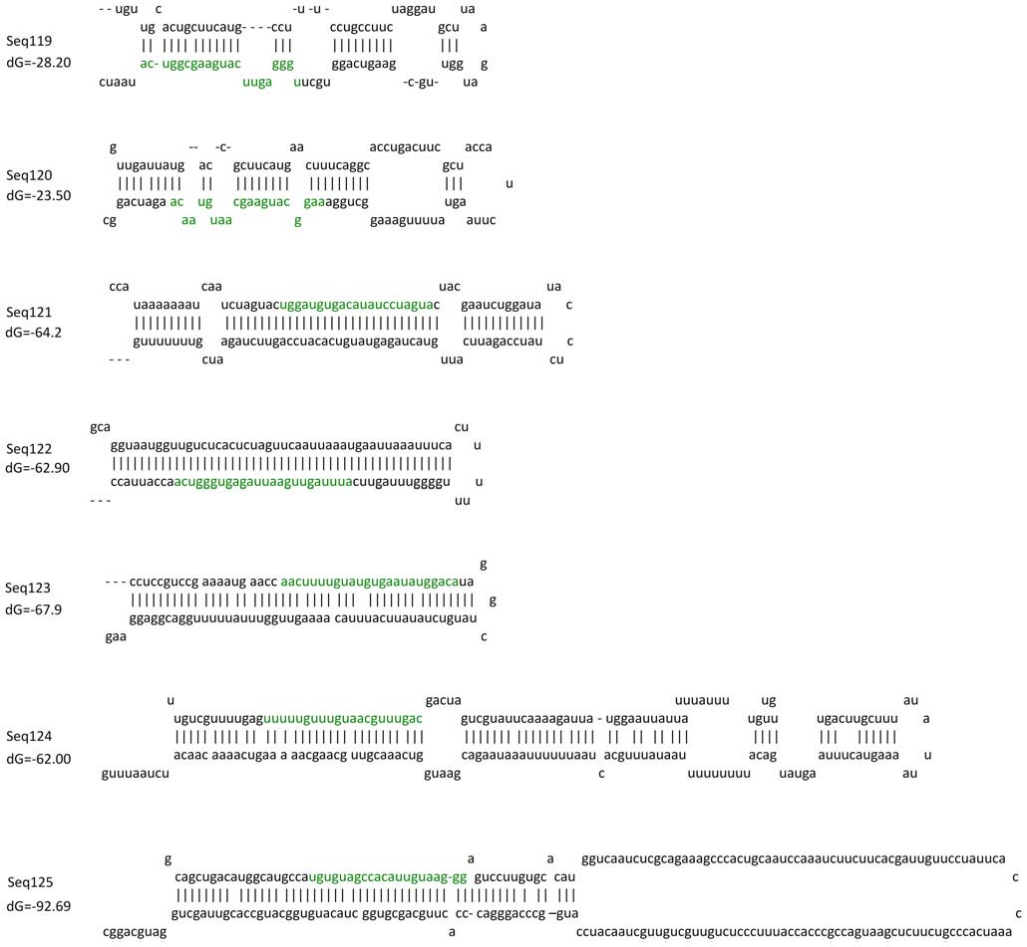
sRNAs that were recognized as pn-miRNAs and the secondary structures of their precursors. The free energy of each structure is indicated as dG (kcal/mol). Sequences of sRNAs are colored green within precursors.

The precursor sequences and corresponding stem-loop structures were predicted for each of the remaining 121 sRNAs that did not match the deposited rice miRNAs or miRNA precursors. 7 sRNAs (Seq119–125) that have precursor sequences capable of forming a stem-loop structure with free energies ranging from −23.5 to −67.9 kcal/mol, and that have no more than 26 hit loci in the rice genome were identified (Table 3, Figure 4), while the other 114 sRNAs had no precursor sequence that could form a stem-loop structure (Table 3). Among these 7 sRNAs, Seq125 had 23 loci in the rice genome and the other sRNAs had only one hit locus each.

**Table 3:** Putative novel miRNAs produced from potential new precursor sequences not previously described.

sRNA No.	length	sRNA sequence	hits No. to rice genome	Location	SR^a^ in exp1^b^	SR in exp2^c^	SR in exp3^c^	SR in exp4^c^	Mean (i/n)^d^	SD	t (3DF)	Significance (alpha)
Seq119	21	UGGGAGUUCAUGAAGCGGUCA	1	intergenic	9992 (10464)	3754 (5517)	35 (498)	50 (668)	0.45	0.445	−2.50	0.088
Seq120	21	AAGGCAUGAAGCAAUGUAACA	1	Antisense to coding gene	779 (727)	1503 (856)	56 (60)	73 (31)	1.53	0.658	1.61	0.206
Seq121	21	UGGAUGUGACAUACUCUAGUA	1	Antisense to coding gene	563 (556)	126 (226)	15 (447)	22 (711)	0.41	0.473	−2.50	0.088
Seq122	24	AUUUAGUUGAAUUAGAGUGGGUCA	1	intergenic	268 (230)	16 (33)	6 (34)	6 (42)	0.49	0.474	−2.14	0.122
Seq123	24	AACUUUUGUAUGUGAAUAUGGACA	1	intergenic	126 (150)	12(25)	8 (48)	5 (67)	0.39	0.346	−3.52	0.039*
Seq124	21	UUUUUGUUUGUAACGUUUGAC	23	intergenic	236 (141)	10 (8)	1 (7)	1 (11)	0.79	0.796	−0.53	0.633
Seq125	21	UGUGUAGCCACAUUGUAAGGG	1	intergenic	141 (110)	4 (5)	20 (77)	21 (117)	0.63	0.514	−1.44	0.246

a: Sequencing reads (SR) were normalized to one million with the unique sequence reads of each library.

b: exp1 is the experiment reported by us. Sequencing reads in the non-infected sRNA library are bracketed, while those in the RSV-infected sRNA library are not.

c: exp2-4 are the three repeats of Du et al (2011). Sequencing reads in non-infected sRNA libraries are bracketed, while those in RSV-infected sRNA libraries are not.

d: mean of the number of infected divided by number of non-infected reads; the following columns show the standard deviation and the ***t-***test value to test whether the mean differs significantly from 1 (equal numbers of reads).

* : indicating the sRNAs that have the significant changes in RSV-infected rice (*p* value<0.05).

All 7 sRNAs could also be found with 1–5517 sequencing reads in the non-infected sRNA library reported by us and in 6 rice sRNA libraries reported by Du et al [38]. The relative high expression level, the stable precursor structures and their stable appearance in rice sRNAs libraries indicated that the 7 sRNAs might be putative novel miRNAs (pn-miRNAs).

### Detection of the pn-miRNAs by Northern blots and Reverse transcription (RT)-PCR

**Figure 5:**
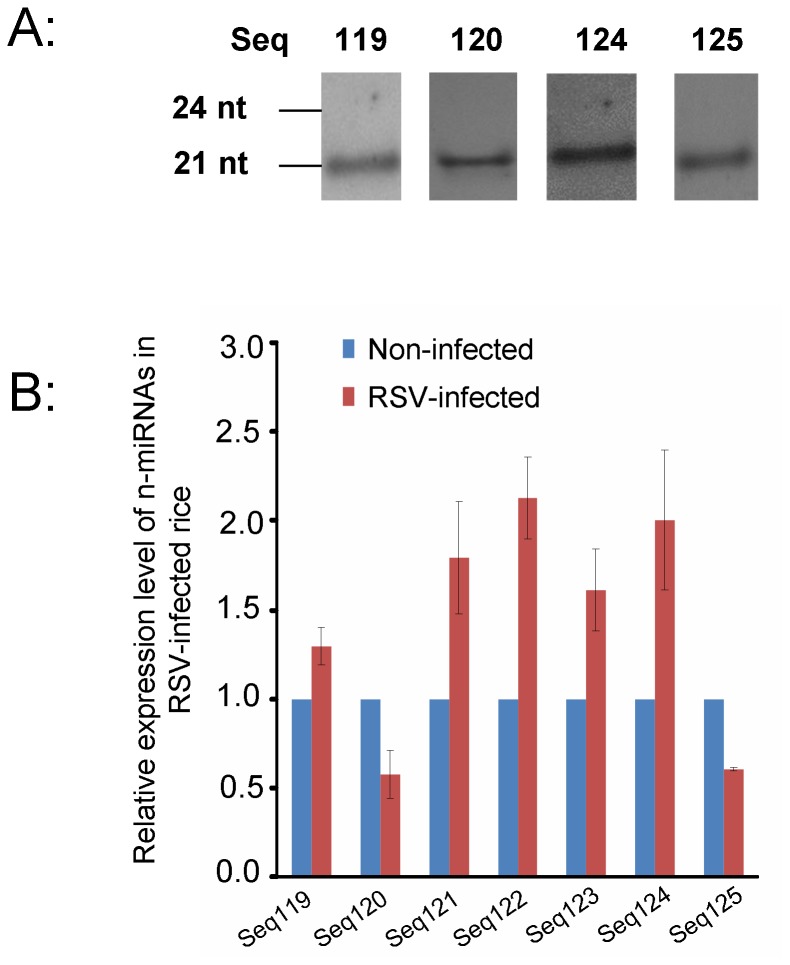
Transcription analyses of n-miRNAs and their changed expression in RSV-infected rice. A, 4 of 7 pn-miRNAs were detectable in Northern blot analysis. B represents the expression analysis of 7 n-miRNAs in RSV-infected rice and non-infected rice through real-time PCR. Expression levels of the 7 n-miRNAs in noninfected rice were assigned as 1.0.

To confirm that the 7 pn-miRNAs were actually produced in plants, we detected them in RSV-infected rice by Northern blots. In three independent experimental repeats, 4 of the 7 pn-miRNAs (Seq119, 120, 124, and 125) could be detected in total RNAs of RSV-infected rice, but the other three pn-miRNAs could not (Figure 5A). Considering the low resolving power of Northern blots, we then detected the pn-miRNAs by RT-PCR. All 7 pn-miRNAs were cloned and their sequences were verified (Figure S1). These results experimentally demonstrate that the 7 pn-miRNAs are actually produced in RSV-infected rice, and can be recognized as novel miRNAs (n-miRNAs).

### Expression analysis of the n-miRNAs in RSV-infected rice

The 7 n-miRNAs analyzed here were identified from the RSV-infected rice sRNA library. To know whether their expression levels were affected by RSV infection, we tried to compare the number of sequencing reads in our reported RSV-infected and non-infected rice sRNA libraries(experiment1, exp1 in table 3) [39], and in the three pairs of RSV-infected and non-infected rice sRNA libraries recently reported by Du et al. (three replicates, exp2–4 in table 3) [38]. The statistical analysis showed only that Seq123 was down-expressed with significant changes in RSV-infected rice, while there were no statistically significant changes for the other n-miRNAs (Table 3). This may reflect the big differences between the four experiments and indicates the fallibility of results taken only from sequencing reads.

To investigate this further, we checked the expression changes in RSV-infected and non-infected rice by real-time PCR. In three independent repeats, Seq120 and Seq125 were down-expressed in RSV-infected rice, while Seq119, Seq121, Seq122, Seq123 and 124 were up-expressed after RSV infection (Figure 5B).

### Predicted targets of the n-miRNAs

The targets of n-miRNAs were predicted by using the web-based psRNA Target Server (http://plantgrn.noble.org/psRNATarget/). Considering that plant miRNA target sites are predominantly located in ORFs, we here only focused on finding target sites in coding regions. Seq119, 120, 121 and 123 had one or two targets, while Seq122, 124 and 125 had three or more targets each (Table 4). Predicted targets were involved in gene expression, signal transduction, energy metabolism and even resistance to stress. The broad range of targets suggests that the identified n-miRNAs play roles during rice development. Moreover, two putative disease resistant proteins were targets of Seq124, suggesting a potential function during RSV infection

**Table 4:** Predicted targets for the identified n-miRNAs in rice.

pn-miRNAs No.	Target Acc.	Maximum expectation	Target Description
Seq119	EE591526	1.0	hypothetical protein
Seq120	NC_008405	3.0	Glycosyl transferase, group 1 domain containing protein
Seq121	CF953168	3.0	Transposon protein
	CI534848	3.0	Transposon protein
Seq122	CI316474	2.0	Predicted protein
	CA762086	2.5	Os11g0311300 protein
	CT862337	3.0	Hatching enzyme
Seq123	CK050672	3.0	Os06g0677700 protein
Seq124	CI455154	1.0	Similar to SEC1-family transport protein SLY1 (AtSLY1)
	CT848706	1.5	Conserved hypothetical protein
	CI309171	1.5	TGF-beta receptor
	EG712217	1.5	ABC transporter permease protein
	CI736378	1.5	LMBR1-like conserved region domain containing protein
	CI247402	2.0	CHCH domain containing protein
	CI276061	2.0	2OG-Fe(II) oxygenase domain containing protein
	CA754682	2.0	Protein of unknown function DTF516 family protein
	CT855745	2.5	Conserved hypothetical protein
	CI317745	2.5	Disease resistance protein family protein
	AK067 489	2.5	Disease resistance protein family protein
Seq125	FG954690	3.0	Mitochondrial carrier protein
	CI741022	3.0	Conserved hypothetical protein
	CA763859	3.0	Mitochondrial carrier protein
	CR281443	3.0	Similar to ATP sulfurylase

## Discussion

The challenge for identification of the novel miRNAs is to judge whether sRNAs satisfy the criteria of miRNAs or not. Generally, three criteria are considered: 1) the miRNA and antisense miRNA should be derived from the opposite stem arms of the precursor stem-loop structure; 2) the mismatched bases between the miRNA and anti-sense miRNA are restricted to four or fewer; and 3) the frequency of asymmetric bulges in miRNA/miRNA* duplex is restricted to less than one and the size should be less than two bases [45]. Several ancillary criteria: conservation, targets, DCL1 dependence and RNA-dependent RNA polymerase (RDR) independence of sRNAs, are also considered when annotating miRNAs, but are not required [45]. Here, 23 pn-miRNAs that were newly produced from known miRNA precursors satisfy the three primary criteria, supporting the case that these 23 are true miRNAs. However, among the 7 pn-miRNAs that were potentially produced from the new precursors, Seq119 and 120 did not satisfy the second and third criteria. Seq 119 had six mismatched bases and Seq120 had five mismatched bases, Moreover, Seq120 had an asymmetric bulge with four bases in the miRNA/miRNA* duplex. However, in the miRBase, not all of the known miRNAs are satisfying all of the criteria. For example, the known osa-miR419 has 14 mismatched bases; osa-miR415 has 10 mismatched bases; osa-miR416 has 7 mismatched bases; osa-miR414 has 7 mismatched bases; osa-miR159a.2 has 6 mismatched bases; osa-miR413 has 6 mismatched bases; osa-miR172a has 5 mismatched bases; osa-miR319a-5p has 5 mismatched bases. Moreover, osa-miR419 has an asymmetric bulge with 7 bases in the miRNA/miRNA* duplex; osa-miR416 has an asymmetric bulge with 6 bases in the miRNA/miRNA* duplex. Hence, it is possible that an sRNA which does not satisfy the three primary criteria is nevertheless a miRNA. Since Seq119 and 120 have precursors with a stable secondary structure, and have potential targets, we recognized them as pn-miRNAs, although they do not satisfy the second and third primary criteria.

In the pyrosequenced sRNA libraries, small interfering RNA from double stranded RNAs or decay products of large precursors are usually found at low frequency [45]. We supposed that siRNA without function might not be specifically accumulated. Hence, to further decrease the effect of siRNA on miRNA identification, we only chose the highly abundant sRNAs with a minimum of 100 sequencing reads in the library for identification, while limiting the locus numbers of sRNAs in the rice genome. This largely eliminates the side effect of siRNA on identification. However, under such conditions, some novel miRNAs present in low abundance might be excluded and it is therefore probable that some further novel miRNAs remain to be discovered.

Deep sequencing has been a powerful method for identifying miRNAs in many plants. In addition, the sequencing reads are thought to reflect the levels of expression of the sequences. Among the n-miRNAs identified here, results from sequencing reads showed that only Seq123 was down-expressed with significant changes in RSV-infected rice. However, real-time PCR results showed that all n-miRNAs had altered expression patterns in the RSV-infected rice: Seq120 and Seq125 were down-expressed in RSV-infected rice, while Seq119, Seq121, Seq122, Seq123 and 124 were up-expressed after RSV infection (Figure 5B). Some of this inconsistency may be because the time after RSV infection when the rice was sampled differed between our research and that of Du et al. We harvested the RSV-infected rice one week after inoculation when RSV infection was systemic, but before symptoms had appeared. In the work reported by Du et al, rice plants were harvested after three weeks when typical symptoms of RSV had appeared. If the expression levels of different n-miRNAs kept changing as the RSV infection developed, such inconsistency is easily explained. It would therefore be interesting to investigate further the exact responses of the n-miRNAs and their functions during the whole process of RSV infection.

In the 46 known miRNA families identified, osa-miR167, 168, 528, 172, 444, 166, 535, 164, 396 and 827 were the ten most abundant miRNA families. This is nearly completely consistent with the results of Du et al., in which osa-miR168, 156, 396, 159, 535, 166, 172, 167, 528 and 444 were the ten most abundant miRNA families, indicating stable functions for these miRNAs in RSV-infected rice. Production of new miRNAs from known precursors was also detected here. One or more new miRNAs were identified from each of 19 known miRNAs. Among these 19 known miRNA precursors, these are the first reports of a second mature miRNA from osa-MIR169a, 187a, 1883b, 1884a, 806g, 807b, 808, 809b, 812a, 1439, 812k and 2123c. Most of the newly produced miRNAs have predicted targets, indicating their potential function in rice (Table S3). Moreover, sequencing reads of Seq99, 102, 104, 105, 107 and 116 showed a stable change between RSV-infected and non-infected rice sRNA libraries, indicating a potential role in RSV-induced rice disease.

RSV-induced rice disease is serious in China, Japan and Korea. Virus variation and the functions of the RSV-encoded proteins have been well studied in recent years [40], [46], [47], [48], [49], [50], [51], [52]. Now we report 7 novel miRNAs related to RSV infection. This is the first report of novel miRNAs related to viral infection in rice. miRNAs play key roles during rice development and contribute to the plant defense against several bacterial, fungal or viral diseases. We are not sure whether the novel miRNAs identified here play some roles in plant defense against viral infection. However, it was found that Seq124 had two potential targets that were putative disease resistance proteins, indicating the possibility that these novel miRNAs function in the rice plant defense against RSV. We intend to do further work to clarify the roles of these novel miRNAs during RSV infection.

## Supporting Information

Figure S1 Alignments of each n-miRNA and its cloned sequence. Sequence 1 represents the n-miRNA sequence; Sequence 2 represents the cloned sequence in pGEM-T vector for sequencing, Sequence 3 represents the expected sequence for cloning.
(TIF)

Table S1 sRNAs used for analysis here.
(XLS)

Table S2 Primers used for RT-PCR and real-time PCR
(DOC)

Table S3 Predicted targets for the identified miRNAs newly produced from known miRNA precursors.
(DOC)

## References

[1] Voinnet O (2009) Origin, biogenesis, and activity of plant microRNAs. Cell 136: 669–687. 19239888 10.1016/j.cell.2009.01.046

[2] Carrington JC, Ambros V (2003) Role of microRNAs in plant and animal development. Science 301: 336–338. 12869753 10.1126/science.1085242

[3] Llave C, Xie Z, Kasschau KD, Carrington JC (2002) Cleavage of Scarecrow-like mRNA targets directed by a class of Arabidopsis miRNA. Science 297: 2053–2056. 12242443 10.1126/science.1076311

[4] Reinhart BJ, Weinstein EG, Rhoades MW, Bartel B, Bartel DP (2002) MicroRNAs in plants. Genes Dev 16: 1616–1626. 12101121 10.1101/gad.1004402 PMC186362

[5] Bartel DP (2004) MicroRNAs: genomics, biogenesis, mechanism, and function. Cell 116: 281–297. 14744438 10.1016/s0092-8674(04)00045-5

[6] Khvorova A, Reynolds A, Jayasena SD (2003) Functional siRNAs and miRNAs exhibit strand bias. Cell 115: 209–216. 14567918 10.1016/s0092-8674(03)00801-8

[7] Wang JW, Wang LJ, Mao YB, Cai WJ, Xue HW, et al (2005) Control of root cap formation by MicroRNA-targeted auxin response factors in Arabidopsis. Plant Cell 17: 2204–2216. 16006581 10.1105/tpc.105.033076 PMC1182483

[8] Palatnik JF, Allen E, Wu X, Schommer C, Schwab R, et al (2003) Control of leaf morphogenesis by microRNAs. Nature 425: 257–263. 12931144 10.1038/nature01958

[9] Kidner CA, Martienssen RA (2005) The developmental role of microRNA in plants. Curr Opin Plant Biol 8: 38–44. 15653398 10.1016/j.pbi.2004.11.008

[10] Jones-Rhoades MW, Bartel DP, Bartel B (2006) MicroRNAS and their regulatory roles in plants. Annu Rev Plant Biol 57: 19–53. 16669754 10.1146/annurev.arplant.57.032905.105218

[11] Guo HS, Xie Q, Fei JF, Chua NH (2005) MicroRNA directs mRNA cleavage of the transcription factor NAC1 to downregulate auxin signals for arabidopsis lateral root development. Plant Cell 17: 1376–1386. 15829603 10.1105/tpc.105.030841 PMC1091761

[12] Chen X (2004) A microRNA as a translational repressor of APETALA2 in Arabidopsis flower development. Science 303: 2022–2025. 12893888 10.1126/science.1088060 PMC5127708

[13] Simon-Mateo C, Garcia JA (2006) MicroRNA-guided processing impairs Plum pox virus replication, but the virus readily evolves to escape this silencing mechanism. J Virol 80: 2429–2436. 16474149 10.1128/JVI.80.5.2429-2436.2006 PMC1395392

[14] Li F, Pignatta D, Bendix C, Brunkard JO, Cohn MM, et al (2011) MicroRNA regulation of plant innate immune receptors. Proc Natl Acad Sci U S A 109: 1790–1795. 10.1073/pnas.1118282109 PMC3277104 22307647

[15] Padmanabhan C, Zhang X, Jin H (2009) Host small RNAs are big contributors to plant innate immunity. Curr Opin Plant Biol 12: 465–472. 19608454 10.1016/j.pbi.2009.06.005

[16] Lu YD, Gan QH, Chi XY, Qin S (2008) Roles of microRNA in plant defense and virus offense interaction. Plant Cell Rep 27: 1571–1579. 18626646 10.1007/s00299-008-0584-z

[17] Navarro L, Dunoyer P, Jay F, Arnold B, Dharmasiri N, et al (2006) A plant miRNA contributes to antibacterial resistance by repressing auxin signaling. Science 312: 436–439. 16627744 10.1126/science.1126088

[18] Kozomara A, Griffiths-Jones S (2011) miRBase: integrating microRNA annotation and deep-sequencing data. Nucleic Acids Res 39: D152–157. 21037258 10.1093/nar/gkq1027 PMC3013655

[19] Fahlgren N, Howell MD, Kasschau KD, Chapman EJ, Sullivan CM, et al (2007) High-throughput sequencing of Arabidopsis microRNAs: evidence for frequent birth and death of MIRNA genes. PLoS One 2: e219. 17299599 10.1371/journal.pone.0000219 PMC1790633

[20] Lu S, Sun YH, Shi R, Clark C, Li L, et al (2005) Novel and mechanical stress-responsive MicroRNAs in Populus trichocarpa that are absent from Arabidopsis. Plant Cell 17: 2186–2203. 15994906 10.1105/tpc.105.033456 PMC1182482

[21] Rajagopalan R, Vaucheret H, Trejo J, Bartel DP (2006) A diverse and evolutionarily fluid set of microRNAs in Arabidopsis thaliana. Genes Dev 20: 3407–3425. 17182867 10.1101/gad.1476406 PMC1698448

[22] Yu X, Wang H, Lu Y, de Ruiter M, Cariaso M, et al (2011) Identification of conserved and novel microRNAs that are responsive to heat stress in Brassica rapa. J Exp Bot 63: 1025–1038. 22025521 10.1093/jxb/err337 PMC3254694

[23] Pelaez P, Trejo MS, Iniguez LP, Estrada-Navarrete G, Covarrubias AA, et al (2012) Identification and characterization of microRNAs in Phaseolus vulgaris by high-throughput sequencing. BMC Genomics 13: 83. 22394504 10.1186/1471-2164-13-83 PMC3359237

[24] Zhao CZ, Xia H, Frazier TP, Yao YY, Bi YP, et al (2010) Deep sequencing identifies novel and conserved microRNAs in peanuts (Arachis hypogaea L.). BMC Plant Biol 10: 3. 20047695 10.1186/1471-2229-10-3 PMC2826338

[25] Lertpanyasampatha M, Gao L, Kongsawadworakul P, Viboonjun U, Chrestin H, et al.. (2012) Genome-wide analysis of microRNAs in rubber tree (Hevea brasiliensis L.) using high-throughput sequencing. Planta. 10.1007/s00425-012-1622-1 PMC3405184 22407387

[26] Pantaleo V, Szittya G, Moxon S, Miozzi L, Moulton V, et al (2010) Identification of grapevine microRNAs and their targets using high-throughput sequencing and degradome analysis. Plant J 62: 960–976. 20230504 10.1111/j.0960-7412.2010.04208.x

[27] Sunkar R, Zhou X, Zheng Y, Zhang W, Zhu JK (2008) Identification of novel and candidate miRNAs in rice by high throughput sequencing. BMC Plant Biol 8: 25. 18312648 10.1186/1471-2229-8-25 PMC2292181

[28] Zhang J, Xu Y, Huan Q, Chong K (2009) Deep sequencing of Brachypodium small RNAs at the global genome level identifies microRNAs involved in cold stress response. BMC Genomics 10: 449. 19772667 10.1186/1471-2164-10-449 PMC2759970

[29] Yao Y, Guo G, Ni Z, Sunkar R, Du J, et al (2007) Cloning and characterization of microRNAs from wheat (Triticum aestivum L.). Genome Biol 8: R96. 17543110 10.1186/gb-2007-8-6-r96 PMC2394755

[30] Mica E, Piccolo V, Delledonne M, Ferrarini A, Pezzotti M, et al (2009) High throughput approaches reveal splicing of primary microRNA transcripts and tissue specific expression of mature microRNAs in Vitis vinifera. BMC Genomics 10: 558. 19939267 10.1186/1471-2164-10-558 PMC2822795

[31] Schreiber AW, Shi BJ, Huang CY, Langridge P, Baumann U (2012) Discovery of barley miRNAs through deep sequencing of short reads. BMC Genomics 12: 129. 10.1186/1471-2164-12-129 PMC3060140 21352554

[32] Song QX, Liu YF, Hu XY, Zhang WK, Ma B, et al (2011) Identification of miRNAs and their target genes in developing soybean seeds by deep sequencing. BMC Plant Biol 11: 5. 21219599 10.1186/1471-2229-11-5 PMC3023735

[33] Chen CJ, liu Q, Zhang YC, Qu LH, Chen YQ, et al (2011) Genome-wide discovery and analysis of microRNAs and other small RNAs from rice embryogenic callus. RNA Biol 8: 538–547. 21525786 10.4161/rna.8.3.15199

[34] Li T, Li H, Zhang YX, Liu JY (2011) Identification and analysis of seven HO-responsive miRNAs and 32 new miRNAs in the seedlings of rice (Oryza sativa L. ssp. indica). Nucleic Acids Res 39: 2821–2833. 21113019 10.1093/nar/gkq1047 PMC3074118

[35] Peng T, Lv Q, Zhang J, Li J, Du Y, et al (2011) Differential expression of the microRNAs in superior and inferior spikelets in rice (Oryza sativa). J Exp Bot 62: 4943–4954. 21791435 10.1093/jxb/err205

[36] Wei LQ, Yan LF, Wang T (2011) Deep sequencing on genome-wide scale reveals the unique composition and expression patterns of microRNAs in developing pollen of Oryza sativa. Genome Biol 12: R53. 21679406 10.1186/gb-2011-12-6-r53 PMC3218841

[37] Wang HD, Chen JP, Zhang HM, Sun XL, Zhu JL, et al (2008) Recent Rice stripe virus Epidemics in Zhejiang Province, China, and Experiments on Sowing Date, Disease-Yield Loss Relationships, and Seedling Susceptibility. Plant Dis 92: 1190–1196. 10.1094/PDIS-92-8-1190 30769483

[38] Du P, Wu J, Zhang J, Zhao S, Zheng H, et al (2011) Viral infection induces expression of novel phased microRNAs from conserved cellular microRNA precursors. PLoS Pathog 7: e1002176. 21901091 10.1371/journal.ppat.1002176 PMC3161970

[39] Yan F, Zhang H, Adams MJ, Yang J, Peng J, et al (2010) Characterization of siRNAs derived from rice stripe virus in infected rice plants by deep sequencing. Arch Virol 155: 935–940. 20396917 10.1007/s00705-010-0670-8

[40] Zhang HM, Yang J, Sun HR, Xin X, Wang HD, et al (2007) Genomic analysis of rice stripe virus Zhejiang isolate shows the presence of an OTU-like domain in the RNA1 protein and a novel sequence motif conserved within the intergenic regions of ambisense segments of tenuiviruses. Arch Virol 152: 1917–1923. 17585367 10.1007/s00705-007-1013-2

[41] Chen C, Ridzon DA, Broomer AJ, Zhou Z, Lee DH, et al (2005) Real-time quantification of microRNAs by stem-loop RT-PCR. Nucleic Acids Res 33: e179. 16314309 10.1093/nar/gni178 PMC1292995

[42] Dai X, Zhao PX (2011) psRNATarget: a plant small RNA target analysis server. Nucleic Acids Res 39: W155–159. 21622958 10.1093/nar/gkr319 PMC3125753

[43] Guo L, Lu Z (2010) Global expression analysis of miRNA gene cluster and family based on isomiRs from deep sequencing data. Comput Biol Chem 34: 165–171. 20619743 10.1016/j.compbiolchem.2010.06.001

[44] Ebhardt HA, Fedynak A, Fahlman RP (2010) Naturally occurring variations in sequence length creates microRNA isoforms that differ in argonaute effector complex specificity. Silence 1: 12. 20534119 10.1186/1758-907X-1-12 PMC2901367

[45] Meyers BC, Axtell MJ, Bartel B, Bartel DP, Baulcombe D, et al (2008) Criteria for annotation of plant MicroRNAs. Plant Cell 20: 3186–3190. 19074682 10.1105/tpc.108.064311 PMC2630443

[46] Zhang C, Pei X, Wang Z, Jia S, Guo S, et al (2012) The Rice stripe virus pc4 functions in movement and foliar necrosis expression in Nicotiana benthamiana. Virology 425: 113–121. 22305130 10.1016/j.virol.2012.01.007

[47] Yao M, Zhang T, Zhou T, Zhou Y, Zhou X, et al (2012) Repetitive prime-and-realign mechanism converts short capped RNA leaders into longer ones that may be more suitable for elongation during rice stripe virus transcription initiation. J Gen Virol 93: 194–202. 21918010 10.1099/vir.0.033902-0

[48] Yuan Z, Chen H, Chen Q, Omura T, Xie L, et al (2011) The early secretory pathway and an actin-myosin VIII motility system are required for plasmodesmatal localization of the NSvc4 protein of Rice stripe virus. Virus Res 159: 62–68. 21565229 10.1016/j.virusres.2011.04.023

[49] Du Z, Xiao D, Wu J, Jia D, Yuan Z, et al (2011) p2 of rice stripe virus (RSV) interacts with OsSGS3 and is a silencing suppressor. Mol Plant Pathol 12: 808–814. 21726383 10.1111/j.1364-3703.2011.00716.x PMC6640460

[50] Wei TY, Yang JG, Liao FL, Gao FL, Lu LM, et al (2009) Genetic diversity and population structure of rice stripe virus in China. J Gen Virol 90: 1025–1034. 19264655 10.1099/vir.0.006858-0

[51] Xiong R, Wu J, Zhou Y, Zhou X (2008) Identification of a movement protein of the tenuivirus rice stripe virus. J Virol 82: 12304–12311. 18818319 10.1128/JVI.01696-08 PMC2593352

[52] Xiong R, Wu J, Zhou Y, Zhou X (2009) Characterization and subcellular localization of an RNA silencing suppressor encoded by Rice stripe tenuivirus. Virology 387: 29–40. 19251298 10.1016/j.virol.2009.01.045

