# Supplementary figures and images for: Identification of Novel Oryza sativa miRNAs in Deep Sequencing-Based Small RNA Libraries of Rice Infected with Rice Stripe Virus
Source: PLoS One. 2012 Oct 10;7(10):e46443. doi: 10.1371/journal.pone.0046443 (PMC3468594; doi:10.1371/journal.pone.0046443)

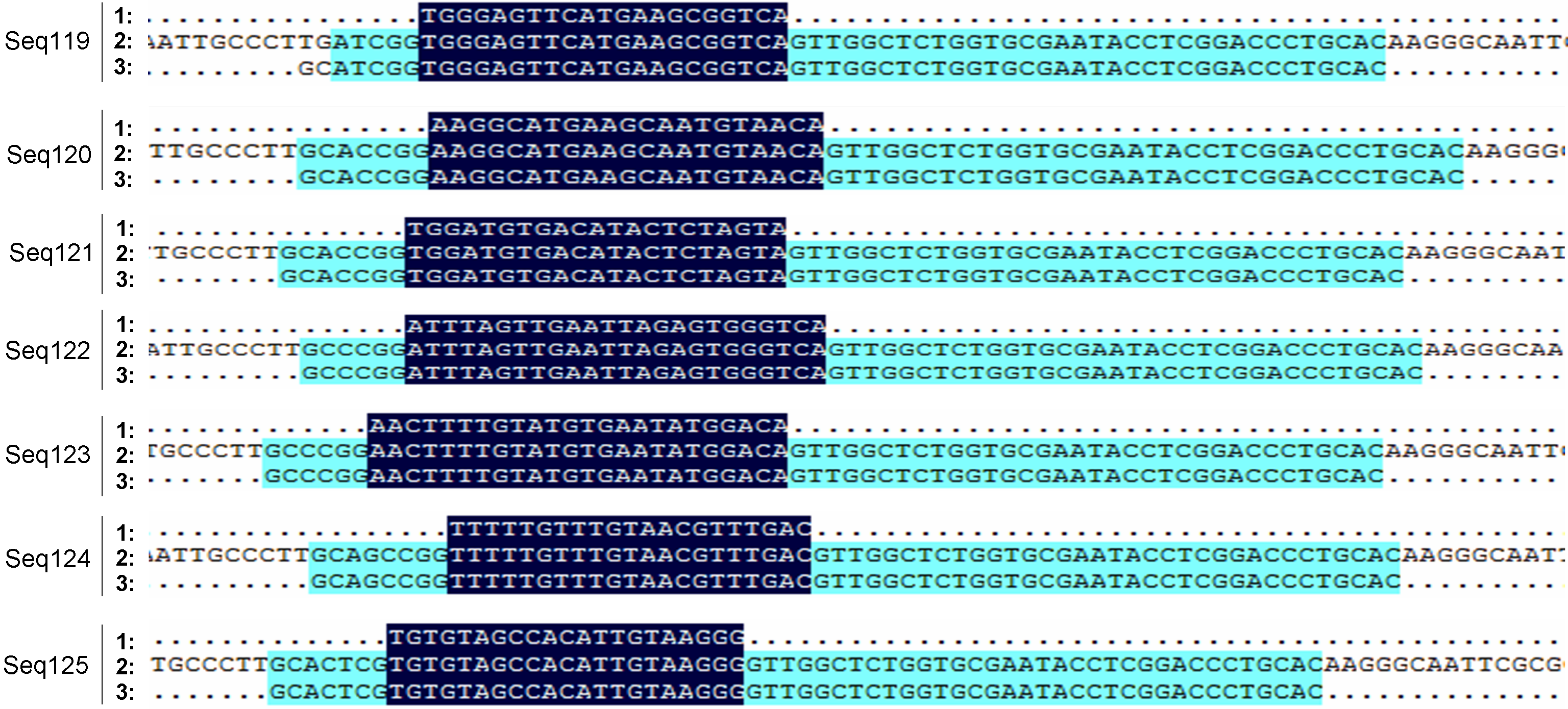

Supplement: Figure S1 — Alignments of each n-miRNA and its cloned sequence. Sequence 1 represents the n-miRNA sequence; Sequence 2 represents the cloned sequence in pGEM-T vector for sequencing, Sequence 3 represents the expected sequence for cloning. (TIF) [file pone.0046443.s001.tif]
