# Supplementary material for: Identification of Novel Oryza sativa miRNAs in Deep Sequencing-Based Small RNA Libraries of Rice Infected with Rice Stripe Virus
Source: PLoS One. 2012 Oct 10;7(10):e46443. doi: 10.1371/journal.pone.0046443 (PMC3468594; doi:10.1371/journal.pone.0046443)
Supplement: Table S2 — Primers used for RT-PCR and real-time PCR (DOC) [file pone.0046443.s003.doc]

| Seq119 | sRNA sequences: | UGGGAGUUCAUGAAGCGGUCA |
| --- | --- | --- |
|  | RT primer: | GTTGGCTCTGGTGCAGGGTCCGAGGTATTCGCACCAGAGCCA ACTGACCG |
|  | Forward primer: | GCATCGGTGGGAGTTCATGAAG |
| Seq120 | sRNA sequences: | AAGGCAUGAAGCAAUGUAACA |
|  | RT primer: | GTTGGCTCTGGTGCAGGGTCCGAGGTATTCGCACCAGAGCCA ACTGTTAC |
|  | Forward primer: | GCACCGGAAGGCATGAAGCAAT |
| Seq121 | sRNA sequences: | UGGAUGUGACAUACUCUAGUA |
|  | RT primer: | GTTGGCTCTGGTGCAGGGTCCGAGGTATTCGCACCAGAGCCA ACTACTAG |
|  | Forward primer: | GCACCGGTGGATGTGACATACT |
| Seq122 | sRNA sequences: | AUUUAGUUGAAUUAGAGUGGGUCA |
|  | RT primer: | GTTGGCTCTGGTGCAGGGTCCGAGGTATTCGCACCAGAGCCA ACTGACCC |
|  | Forward primer: | GCCCGGATTTAGTTGAATTAGAGT |
| Seq123 | sRNA sequences: | AACUUUUGUAUGUGAAUAUGGACA |
|  | RT primer: | GTTGGCTCTGGTGCAGGGTCCGAGGTATTCGCACCAGAGCCA ACTGTCCA |
|  | Forward primer: | GCCCGGAACTTTTGTATGTGAATA |
| Seq124 | sRNA sequences: | UUUUUGUUUGUAACGUUUGAC |
|  | RT primer: | GTTGGCTCTGGTGCAGGGTCCGAGGTATTCGCACCAGAGCCA ACGTCAAA |
|  | Forward primer: | GCAGCCGGTTTTTGTTTGTAACG |
| Seq125 | sRNA sequences: | UGUGUAGCCACAUUGUAAGGG |
|  | RT primer: | GTTGGCTCTGGTGCAGGGTCCGAGGTATTCGCACCAGAGCCA ACCCCTTA |
|  | Forward primer: | GCACTCGTGTGTAGCCACATTG |
| U6 | Forward primer: | TACAGATAAGATTAGCATGGCCCC |
|  | Reverse primer: | GGACCATTTCTCGATTTGTACGTG |
| Universal | Reverse primer: | GTGCAGGGTCCGAGGT |
