# Supplementary material for: Identification of Novel Oryza sativa miRNAs in Deep Sequencing-Based Small RNA Libraries of Rice Infected with Rice Stripe Virus
Source: PLoS One. 2012 Oct 10;7(10):e46443. doi: 10.1371/journal.pone.0046443 (PMC3468594; doi:10.1371/journal.pone.0046443)
Supplement: Table S3 — Predicted targets for the identified miRNAs newly produced from known miRNA precursors. (DOC) [file pone.0046443.s004.doc]

| pn-miRNAs No. | Target Acc. | Maximum expectation | Target Description |
| --- | --- | --- | --- |
| Seq96 | CI285685 | 2.0 | Small GTP binding protein Rab1A |
|  | CI310105 | 2.5 | Sodium/hydrogen exchanger family protein |
|  | TC460123 | 2.5 | Gigantea protein |
| Seq97 | TC416410 | 3.0 | hypothetical protein |
|  | TC419893 | 3.0 | hypothetical protein |
| Seq98 | CA753388 | 3.0 | Alanyl aminopeptidase. Metallo peptidase. MEROPS family M01 |
| Seq99 | BQ908452 | 2.5 | hypothetical protein |
|  | BQ908470 | 2.5 | Envelope glycoprotein |
|  | TC461392 | 2.5 | Thylakoid lumen protein, chloroplast-like |
|  | NP1351892 | 3.0 | adsorption protein-like |
|  | TC414908 | 3.0 | UDP-glucose:protein transglucosylase-like protein SlUPTG1 - Solanum lycopersicum |
| Seq100 | CX113360 | 3.0 | hypothetical protein |
|  | FG965893 | 3.0 | Peptidyl-prolyl cis-trans isomerase |
| Seq101 | TC420448 | 0.5 | Zinc finger A20 domain-containing stress-associated protein 18 |
|  | TC461168 | 0.5 | Protein kinase-like domain containing protein |
|  | TC461610 | 1.0 | Zinc finger A20 domain-containing stress-associated protein 18 |
|  | CA761694 | 1.0 | CD9/CD37/CD63 antigen family protein |
|  | TC409230 | 1.0 | Glycoside hydrolase, family 1 protein . |
|  | TC406891 | 1.0 | HMG-I and HMG-Y, DNA-binding domain containing protein |
|  | TC422341 | 1.5 | Ankyrin-like protein |
|  | TC439786 | 1.5 | Ankyrin repeat containing protein |
|  | CI135971 | 1.5 | GDP-mannose pyrophosphorylase - Pinus taeda (Loblolly pine) |
|  | CB637008 | 1.5 | Similar to Cell wall invertase (EC 3.2.1.26) |
|  | CI126585 | 1.5 | Similar to Serine/threonine protein kinase |
|  | TC409234 | 1.5 | Hypothetical protein |
|  | TC466227 | 1.5 | Cytochrome P450 family protein Pr1-like protein |
|  | TC437504 | 1.5 | Zinc finger A20 domain-containing stress-associated protein 18 |
|  | TC420642 | 1.5 | Chromosome 04 contig 1, DNA sequence - Ostreococcus tauri |
|  | TC480065 | 2.0 | Conserved hypothetical protein |
|  | CB638005 | 2.0 | Zinc finger, CCCH-type domain containing protein |
|  | TC406205 | 2.0 | Beta-fructofuranosidase, insoluble isoenzyme 3 precursor |
|  | TC463057 | 2.5 | Basic helix-loop-helix dimerisation region bHLH domain containing protein |
|  | CI166102 | 2.5 | Ras family protein - Trichomonas vaginalis G3 |
|  | TC432787 | 2.5 | CD9/CD37/CD63 antigen family protein |
|  | TC451163 | 2.5 | Non-specific lipid transfer protein precursor - Fragaria ananassa (Strawberry) |
|  | CK085301 | 2.5 | Adenine deaminase - Rubrobacter xylanophilus (strain DSM 9941 / NBRC 16129) |
|  | CI749603 | 2.5 | Serine/threonine protein kinase domain containing protein |
|  | TC459105 | 2.5 | UDP-glucuronosyl/UDP-glucosyltransferase family protein cont -ainns InterPro domain |
|  |  |  |  |
| Seq102 | TC405592 | 0.0 | transcription factor IIA gamma subunit |
|  | TC418137 | 0.0 | Protein of unknown function, ATP binding family protein |
|  | TC467503 | 0.0 | Similar to 60S ribosomal protein L10a-3 |
|  | CB676942 | 0.0 | Similar to Probenazole-inducible protein PBZ1 |
|  | TC425671 | 0.0 | Hypothetical protein |
|  | TC473994 | 0.0 | Metallothionein-like protein 4B |
|  | TC449544 | 0.0 | Similar to Receptor kinase-like protein |
|  | TC483544 | 0.0 | Zinc finger A20 domain-containing stress-associated protein 18 |
|  | TC409994 | 0.5 | Leucine-rich repeat, plant specific containing protein |
|  | TC415398 | 0.5 | .UDP-glucuronosyl/UDP-glucosyltransferase family protein |
|  | TC435201 | 0.5 | Cell wall protein pherophorin-C10 |
|  | TC441455 | 0.5 | Conserved hypothetical protein |
|  | TC447545 | 0.5 | Predicted protein - Ajellomyces capsulata (strain NAm1) |
|  | NP656437 | 0.5 | YABBY protein (OsYAB1) (Filamentous flower protein 1)~ |
|  | TC483637 | 0.5 | RasGEF domain containing protein - Trichomonas vaginalis G3 |
|  | CB637008 | 0.5 | Similar to Cell wall invertase (EC 3.2.1.26) |
|  | TC416947 | 1.0 | mitogen-activated protein kinase kinase-like |
|  | CT847545 | 1.0 | Flavin containing monooxygenase 4-like protein |
|  | TC450169 | 1.0 | Carotenoid cleavage dioxyganase 1 - Zea mays (Maize), |
|  | TC454495 | 1.0 | Nrap protein, expressed - Oryza sativa subsp. japonica (Rice), |
|  |  |  |  |
| Seq 103 | TC415590 | 2.5 | Maf-like protein family protein |
|  | CX118396 | 3.0 | Similar to Proline iminopeptidase |
|  | CT857693 | 3.0 | CLE family OsCLE204 protein |
|  |  |  |  |
| Seq 104 | TC409569 | 0.0 | RINGv domain containing protein |
|  | TC409626 | 0.0 | Transcriptional factor B3 family protein |
|  | CI565500 | 0.0 | Glutaredoxin-related protein family protein Monothiol glutared -oxin-S4 precursor |
|  | CI187844 | 0.0 | DOMON related domain containing protein |
|  | TC421011 | 0.0 | Lipase, class 3 family protein |
|  | TC451368 | 0.0 | RINGv domain containing protein |
|  | TC452266 | 0.0 | Conserved hypothetical protein |
|  | TC410436 | 0.0 | Peptidase A1, pepsin family protein |
|  | TC479331 | 0.5 | 3'-5' exonuclease domain containing protein Thioredoxin dom -ain 2; Thioredoxin fold - Medicago truncatula (Barrel medic) |
|  | TC483054 | 0.5 | RRNA intron-encoded homing endonuclease |
|  | TC416214 | 0.5 | Protein farnesyltransferase/ geranylgeranyltransferase type I alpha subunit |
|  | TC417323 | 0.5 | Short-chain dehydrogenase/reductase SDR family protein con -tains InterPro domain |
|  |  |  |  |
| Seq 105 | CB673064 | 0.0 | Conserved hypothetical protein |
|  | TC409569 | 0.0 | RINGv domain containing protein |
|  | TC409626 | 0.0 | Transcriptional factor B3 family protein |
|  | TC421011 | 0.0 | Lipase, class 3 family protein |
|  | CI187844 | 0.0 | DOMON related domain containing protein |
|  | TC451368 | 0.0 | RINGv domain containing protein |
|  | TC416009 | 0.0 | Ras GTPase family protein contains InterPro domain(s) |
|  | TC441816 | 0.0 | Tetratricopeptide-like helical domain containing protein |
|  | TC452266 | 0.0 | Conserved hypothetical protein Os09g0512800 Similar to Neural Wiskott-Aldrich syndrome protein (N-WASP)~ |
|  | TC410436 | 0.0 | Peptidase A1, pepsin family protein |
|  | TC409692 | 0.0 | Protein of unknown function DUF604 family protein |
|  | TC445832 | 0.0 | Conserved hypothetical protein |
|  | TC416214 | 0.5 | Similar to Protein farnesyltransferase/geranylgeranyltransferase type I alpha subunit |
|  | TC417323 | 0.5 | Short-chain dehydrogenase/reductase SDR family protein cont -ains InterPro domain(s) |
|  |  |  |  |
| Seq 106 | TC403448 | 0.0 | Conserved hypothetical protein |
|  | TC409569 | 0.0 | RINGv domain containing protein |
|  | TC409626 | 0.0 | Transcriptional factor B3 family protein |
|  | TC421011 | 0.0 | Lipase, class 3 family protein |
|  | CB673064 | 0.0 | Similar to Aspartic proteinase oryzasin 1 precursor |
|  | TC409692 | 0.0 | Protein of unknown function DUF604 family protein |
|  | TC432135 | 0.0 | Protein of unknown function DUF159 family protein |
|  | CI312170 | 0.0 | Glucose/ribitol dehydrogenase family protein |
|  | TC417323 | 0.0 | Short-chain dehydrogenase/reductase SDR family protein |
|  | TC441816 | 0.0 | Tetratricopeptide-like helical domain containing protein |
|  | CB643658 | 0.0 | Glycoprotein 3-alpha-L-fucosyltransferase |
|  | TC416009 | 0.0 | Ras GTPase family protein |
|  | TC410436 | 0.0 | Peptidase A1, pepsin family protein |
|  | TC445832 | 0.0 | Conserved hypothetical protein |
|  | TC408545 | 0.0 | Lecithin:cholesterol acyltransferase family protein |
|  | CX101641 | 0.0 | Transcriptional factor B3 family protein |
|  | TC472065 | 0.0 | Protein kinase domain containing protein |
|  |  |  |  |
| Seq 107 | CA764487 | 0.0 | Hypothetical protein |
|  | CI285448 | 0.0 | Conserved hypothetical protein |
|  | TC409234 | 0.0 | Hypothetical protein |
|  | TC448500 | 0.0 | Disease resistance protein family protein |
|  | TC426468 | 0.0 | RING zinc finger protein-like |
|  | TC468590 | 0.0 | Hypothetical protein |
|  | TC412746 | 0.0 | Cyclin-like F-box domain containing protein |
|  | CR285300 | 0.0 | Conserved hypothetical protein |
|  | TC414856 | 0.5 | Protein of unknown function DUF1677,Oryza sativa family pro -tein |
|  | TC436440 | 0.5 | Conserved hypothetical protein |
|  | TC444078 | 0.5 | Similar to Nectarin 5 (Fragment) |
|  | TC411463 | 0.5 | Peptidase S10, serine carboxypeptidase family protein |
|  | TC445215 | 0.5 | Probable 6-phosphogluconolactonase 2 |
|  | TC437438 | 1.0 | Nitrogen-fixing NifU-like, N-terminal domain containing protein |
|  | CT852779 | 1.0 | M.musculus 45S pre rRNA gene |
|  | TC422607 | 1.0 | Conserved hypothetical protein |
|  | BQ907311 | 1.0 | Similar to Iron sulfur cluster assembly protein 1, mitochondrial precursor (Iron sulfur cluster scaffold protein 1) |
|  | TC447180 | 1.0 | hypothetical protein |
|  | CA766726 | 1.0 | Mak16 protein family protein |
|  | TC437522 | 1.5 | TPR-like domain containing protein |
|  | TC439883 | 1.5 | GRIM-19 family protein |
|  |  |  |  |
| Seq 108 | CF306131 | 0.0 | Zinc/iron permease family protein |
|  | CI135971 | 0.5 | GDP-mannose pyrophosphorylase - Pinus taeda (Loblolly pine), |
|  | TC433274 | 1.0 | Hypothetical protein |
|  | TC411250 | 1.0 | CBL-interacting protein kinase 10;protein kinase |
|  | CA763969 | 1.5 | PE-PGRS family protein -Mycobacterium tuberculosis |
|  | CA763409 | 1.5 | Conserved hypothetical protein |
|  | TC401890 | 1.5 | Spectrin repeat containing protein |
|  | TC427531 | 1.5 | Glycoside hydrolase, family 17 protein |
|  | CK085301 | 1.5 | Adenine deaminase - Rubrobacter xylanophilus |
|  | TC432787 | 1.5 | CD9/CD37/CD63 antigen family protein |
|  | TC439786 | 1.5 | Ankyrin repeat containing protein |
|  | EX493721 | 1.5 | CD9/CD37/CD63 antigen family protein |
|  | TC453898 | 1.5 | Conserved hypothetical protein |
|  | TC451163 | 1.5 | Non-specific lipid transfer protein precursor - Fragaria ananassa (Strawberry), |
|  | TC431820 | 1.5 | Peptidase A1, pepsin family protein |
|  | TC472313 | 1.5 | CLE family OsCLE204 protein |
|  | TC459105 | 2.0 | UDP-glucuronosyl/UDP-glucosyltransferase family protein |
|  | TC445525 | 2.0 | Conserved hypothetical protein |
|  | TC483276 | 2.0 | UDP-glucuronosyl/UDP-glucosyltransferase family protein |
|  | TC426627 | 2.5 | Expressed protein, having alternative splicing products |
|  | TC403064 | 2.5 | PRIP-interacting protein-like |
|  | CI417415 | 2.5 | Glycoside hydrolase, family 17 protein |
|  |  |  |  |
| Seq 109 | CR281092 | 0.0 | Similar to Amino acid carrier (Fragment) |
|  | TC425401 | 0.5 | Similar to Beta-hexosaminidase beta chain precursor |
|  | TC469599 | 1.0 | Cytochrome P450 71C3 - Zea mays (Maize) |
|  | TC437368 | 1.0 | Similar to Amino acid carrier (Fragment) |
|  | TC442927 | 1.0 | PAP/25A core domain containing protein |
|  | TC436409 | 1.5 | Peptidyl-prolyl cis-trans isomerase - Vitis vinifera (Grape), |
|  | CI414008 | 1.5 | Uncharacterized protein |
|  | CI324158 | 1.5 | Similar to FK506-binding protein 2-2 precursor |
|  | TC403470 | 1.5 | Receptor-like protein kinase 6 |
|  | TC426599 | 1.5 | Cell wall glycoprotein GP2 - Chlamydomonas reinhardtii, |
|  | CI329601 | 1.5 | 2OG-Fe(II) oxygenase domain containing protein |
|  | TC421795 | 2.0 | Thioredoxin family protein |
|  | CT857767 | 2.0 | Conserved hypothetical protein |
|  | TC463172 | 2.0 | Predicted protein - Physcomitrella patens subsp. patens, |
|  |  |  |  |
|  |  |  |  |
| Seq 110 | CI410686 | 0.0 | Similar to Transcription factor (PWWP domain protein)-like pro -tein |
|  | TC444332 | 0.0 | Kelch motif family protein, |
|  | TC404211 | 0.0 | AIG1 family protein |
|  | CV734001 | 0.0 | Galactose oxidase, central domain containing protein |
|  | TC416197 | 0.0 | Calcium-dependent protein kinase CPK1 adapter protein 2-like |
|  | TC412438 | 0.0 | Similar to Protein synthesis inhibitor II |
|  | BQ908022 | 0.0 | Zinc finger A20 domain-containing stress-associated protein 18 |
|  | TC446798 | 0.0 | Similar to Geranylgeranyltransferase beta subunit |
|  | BI812454 | 0.0 | Zinc finger A20 domain-containing stress-associated protein 18 |
|  | CT851791 | 0.5 | Hypothetical protein |
|  | CB637008 | 1.0 | Similar to Cell wall invertase (EC 3.2.1.26) |
|  | CI126585 | 1.0 | Similar to Serine/threonine protein kinase |
|  | TC454054 | 1.5 | Conserved hypothetical protein |
|  | CT859695 | 1.5 | Similar to 1-aminocyclopropane-1-carboxylate oxidase (Fragme -nt) |
|  | TC436785 | 1.5 | Protein kinase domain containing protein |
|  | TC455141 | 1.5 | Conserved hypothetical protein |
|  | TC483569 | 2.0 | Conserved hypothetical protein |
|  | CA765319 | 2.0 | Claudin 1 - Fugu rubripes (Japanese pufferfish) |
|  | TC435936 | 2.5 | 1-aminocyclopropane-1-carboxylate oxidase |
|  | TC418067 | 2.5 | Protein kinase domain containing protein |
|  | TC452908 | 2.5 | ZIM motif family protein, expressed |
|  |  |  |  |
| Seq 111 | TC425584 | 1.0 | Protein of unknown function DUF1637 family protein |
|  | TC424557 | 1.0 | Cellular retinaldehyde-binding/triple function, N-terminal domain containing protein |
|  | TC405352 | 2.0 | ABC transporter, transmembrane region domain containing pro -tein |
|  | TC427068 | 2.0 | Protein of unknown function DUF1399 family protein |
|  | TC413267 | 2.5 | Cyclin-like F-box domain containing protein |
|  |  |  |  |
| Seq 112 | TC401230 | 0.0 | Conserved hypothetical protein |
|  | TC471112 | 0.0 | Similar to RING finger protein 13 (C-RZF) |
|  | CK080501 | 0.5 | E-class P450, group I family protein |
|  | CB649351 | 0.5 | Nucleotide-binding, alpha-beta plait domain containing protein |
|  | TC424888 | 1.0 | Pectinesterase inhibitor domain containing protein |
|  | CX110095 | 1.0 | D5L protein - Variola minor virus |
|  | TC439358 | 1.5 | Hypothetical protein |
|  |  |  |  |
| Seq 113 | CT847545 | 0.0 | Flavin containing monooxygenase 4-like protein |
|  | TC417983 | 0.0 | Similar to Growth-regulating factor 7 |
|  | TC423750 | 0.0 | Conserved hypothetical protein |
|  | TC465005 | 0.0 | Conserved hypothetical protein |
|  | CI740953 | 0.0 | Protein of unknown function DUF300 family protein |
|  | TC446817 | 0.0 | Thymidylate synthase domain containing protein |
|  | TC425671 | 0.0 | Hypothetical protein |
|  | TC467503 | 0.0 | Similar to 60S ribosomal protein L10a-3 |
|  | TC469773 | 0.0 | Epstein-Barr virus EBNA-1-like protein |
|  | NP937496 | 0.0 | hypothetical protein |
|  | TC418137 | 1.0 | Protein of unknown function, ATP binding family protein |
|  | TC443601 | 1.0 | Conserved hypothetical protein |
|  | TC418886 | 1.0 | C2 domain containing protein |
|  | TC426607 | 1.0 | 39 kDa antigen - Leishmania donovani |
|  | TC477333 | 1.5 | Major facilitator superfamily protein |
|  | CI736503 | 1.5 | Hypothetical protein |
|  | TC467900 | 1.5 | Conserved hypothetical protein |
|  | TC411807 | 1.5 | Histone H2B.9 |
|  | TC433920 | 2.0 | Legume lectin, beta domain containing protein |
|  |  |  |  |
| Seq 114 | CR286380 | 2.0 | S-receptor kinase-like |
|  | FG957417 | 2.0 | S-receptor kinase-like |
|  | AA754132 | 2.0 | Conserved hypothetical protein |
|  | TC429668 | 2.5 | Auxin-induced protein - Saccharum hybrid cultivar |
|  | TC462823 | 2.5 | Nt-iaa4.1 deduced protein - Nicotiana tabacum |
|  | TC406212 | 2.5 | Leucine rich repeat, N-terminal domain containing protein |
|  | CB631664 | 3.0 | contains InterPro domain(s) |
|  | TC416604 | 3.0 | Protein kinase-like domain containing protein |
|  |  |  |  |
| Seq 115 | CR286380 | 2.5 | S-receptor kinase-like |
|  | FG957417 | 2.5 | S-receptor kinase-like |
|  | AA754132 | 2.5 | Conserved hypothetical protein |
|  | TC406212 | 3.0 | Leucine rich repeat, N-terminal domain containing protein |
|  |  |  |  |
| Seq116 | TC418421 | 0.0 | Monosiga brevicollis MX1 |
|  | NP653568 | 1.0 | protein kinase-like |
|  | TC404612 | 1.0 | Similar to Pto kinase interactor 1 |
|  | CA756639 | 2.5 | hypothetical protein |
|  | NP435002 | 2.5 | hypothetical protein |
|  | FG954057 | 3.0 | Methylmalonyl-CoA mutase large subunit |
|  | TC443577 | 3.0 | hypothetical protein |
| Seq 117 | TC445428 | 0.0 | Conserved hypothetical protein |
|  | TC427235 | 2.0 | Conserved hypothetical protein |
|  | TC429526 | 3.0 | Dof domain, zinc finger family protein, |
|  |  |  |  |
| Seq118 | TC455386 | 2.5 | BTB domain containing protein |
|  | TC402873 | 3.0 | SAM dependent carboxyl methyltransferase |
|  | TC444635 | 3.0 | hypothetical protein |
|  | CT853930 | 3.0 | Similar to Serine/threonine kinase Os12g0132200 |
|  | TC402765 | 3.0 | Protein of unknown function DUF608 domain containing protein |
|  | TC462197 | 3.0 | Protein of unknown function DUF608 domain containing protein |
